# Systematic Targeted Integration to Study *Albumin* Gene Control Elements

**DOI:** 10.1371/journal.pone.0023234

**Published:** 2011-08-12

**Authors:** Sanchari Bhattacharyya, Jianmin Tian, Eric E. Bouhassira, Joseph Locker

**Affiliations:** 1 Department of Pathology, Albert Einstein College of Medicine, Bronx, New York, United States of America; 2 The Marion Bessin Liver Center, Albert Einstein College of Medicine, Bronx, New York, United States of America; 3 Department of Medicine, Albert Einstein College of Medicine, Bronx, New York, United States of America; University of Florida, United States of America

## Abstract

To study transcriptional regulation by distant enhancers, we devised a system of easilymodified reporter plasmids for integration into single-copy targeting cassettes in clones of HuH7, a human hepatocellular carcinoma. The plasmid constructs tested transcriptional function of a 35-kb region that contained the rat albumin gene and its upstream flanking region. Expression of integrants was analyzed in two orientations, and compared to transient expression of non-integrated plasmids. Enhancers were studied in their natural positions relative to the promoter and localizedby deletion. All constructs were also analyzed by transient transfection assays. In addition to the known albumin gene enhancer (E1 at −10 kb), we demonstrated two new enhancers, E2 at −13, and E4 at +1.2 kb. All three enhancers functioned in both transient assays and integrated constructs. However, chromosomal integration demonstrated several differences from transient expression. For example, analysis of E2 showed that enhancer function within the chromosome required a larger gene region than in transient assays. Another conserved region, E3 at −0.7 kb, functioned as an enhancer in transient assays but inhibited the function of E1 and E2 when chromosomally integrated. The enhancers did not show additive or synergistic behavior,an effect consistent with competition for the promoter or inhibitory interactions among enhancers. Growth arrest by serum starvation strongly stimulated the function of some integrated enhancers, consistent with the expected disruption of enhancer-promoter looping during the cell cycle.

## Introduction

In addition to the core promoter and its proximal regulatory element, many genes have enhancers, which vary in size, location, strength, and mode of action. Ranging from 50–1500 bp, enhancers can activate their target promoters from great distances (up to 1 mb) and may reside within introns or even on different chromosomes [1], [2]. Distant enhancers physically associate with promoters by looping out large intervening sequences [3]. Theselong-range interactions regulate developmental and tissue-specific gene expression. However, the variety of enhancers and the large genomic distances separating them from promoters have hindered mechanistic study of their interactions within chromosomes.

Individual enhancers may vary in promoter specificity and strength, and combinations may additively, synergistically, or competitively stimulate transcription [4], [5], [6]. In addition, many enhancers can activate heterologous weak promoters, a property exploited in *enhancer trapping* [7], [8]. Promoters may compete for enhancers [9], [10], and there are also a few cases where multiple promoters share enhancers [11], [12]. The study of integrated activity in an intact gene therefore goes beyond simple demonstrations of enhancer function.

There has been great progress in finding enhancers. Conserved or ultraconserved regions in non-coding DNA frequently identify enhancers, but do not always correlate with transcriptional function [13]. Active enhancers are marked by monomethylated lysine 4 on histone 3 (H3K4) in contrast to H3K4 trimethylation of active promoters. DNAseI hypersensitive sites, transcription factor binding, and coactivator binding also correlate with enhancer function [3], [14], [15], [16]. Chromatin Conformation Capture and its updated versions have shown physical association of distant regulatory regions to target promoters [17], [18], [19], [20], [21]. These contemporary approaches have predicted 10^5^–10^6^ enhancers in the genome, i.e., an enhancer every 3–30 kb [8]. Nevertheless,the approximate and predictive enhancer localization by these methods is not equivalent to functional assessment [22].

Since the discovery of enhancers, transient assay in reporter plasmids has provided a standard test of function independent of chromosomal context. The limitations of this approach are exemplified by the β-globin *locus control region* (LCR), a complex of enhancers and other functional elements that render position-independence to transgenes [23], [24]. Some LCR components regulatetranscription in transient assays whileothers require integration [8]. However, integration of transgenesis subject tovariable copy number and chromosomal position, which makes it difficult to compare different integrated constructs. Wetherefore derived a system for analyzing sets of single copy gene constructs in a consistent chromosomal position, and then used this system to study the *Albumin* gene.

Prior studies of *Alb*—a classical marker of the liver phenotype—established paradigms for gene expression controlled by distant enhancers. The 16.5-kb rat *Alb* gene has 15 exons and a single liver-specific enhancer (“E1”) at −10 kb, a remarkable distance at the time of its discovery [25]. Most E1 function has been mapped to a 200-bp minimal enhancer that binds FOXA and GATA4 [26], [27], [28], [29], [30]. Nevertheless, E1 only weakly stimulates transcription in transient assays [25], [31]. This weakactivityraises intriguing questions, whether E1 needs integration in its natural context for proper function, and whether additional enhancers are required for optimum *Alb* expression. Our analysis characterizeda 35-kb region containing the full *Alb* gene and 18-kb upstream segment. The region includes E1 and several other conserved non-coding regions, potentially novel regulatory elements.

We adapted an efficient system of *recombinase mediated cassette exchange* (RMCE) that uses inverted LoxP sites to prevent re-excision of the integrated transgene and allows insertion of DNA segments that do not contain a selection marker [32], [33]. After incorporation of a targeting cassette into hepatocyte-like HuH7 cells, integration of *Alb*-gene reporters validated the experimental system by demonstrating that transcription of *Alb* is regulated by a much more complicated system of distant enhancers than revealed by previous studies.

## Results

### Transcriptionally permissive target loci

**Figure 1:**
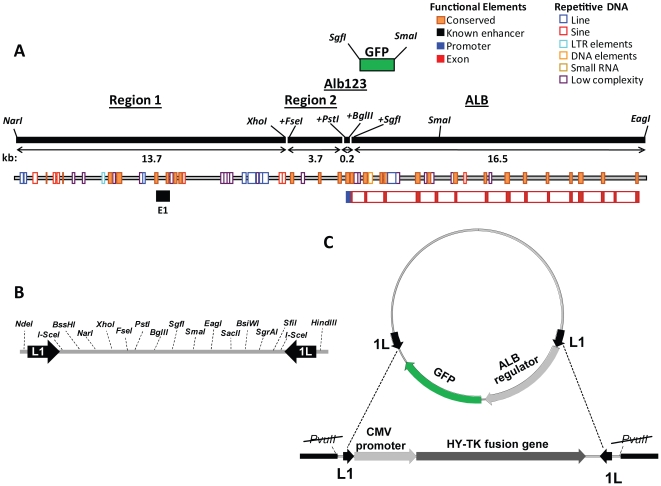
Cloning and recombination strategies. A: Rat *Alb* and its upstream region. Using restriction sites in the pLL1 linker, GFP was combined with Alb123, Region 1 (a *NarI – XhoI* segment from −13.7 to −3.9 kb), Region 2 (a segment from −3.9 to −0.2 kb, generated by PCR that added terminal *FseI* and *PstI* sites), and Alb123 (−0.2 to the transcription start, with terminal *PstI* and *BglII* sites) in a series of reporter plasmids. The 16.5-kb *Alb* gene was cloned by joining a 4.4 kb proximal segment (transcription start to *SmaI*, amplified by PCR that added an *SgfI* site) and a 12.1 kb distal segment (*SmaI* – *EagI*). To insert the Alb123-GFP reporter as an *FseI*–*SmaI* segment, the *Alb*-containing plasmid was cut with *FseI* and *SgfI*, and blunted at the latter site. The resulting reporter plasmids ranged from 4–40 kb (Table S1). B: Schematic map of the 290 bp linker showing the restriction sites for locus assembly. C: Mechanism of RMCE. The targeting cassette encodes an HY-TK fusion protein that makes the cell sensitive to Gancyclovir. Integration of reporter constructs in pLL1 or pLL2 occurs via Cre-mediated recombination, and can take place in two orientations due to the inverted arrangement of the LoxP sites [32]. Loss of the HY-TK gene renders the integrant cells resistant to Gancyclovir.

We used human HuH7 cells to study *Alb* transcription controls because they express high levels of Alb mRNA like fetal and adult hepatocytes [34]. HuH7 cell clones with chromosomally integrated targeting cassettes were isolated by hygromycin selection after transfection. Southern blot identified clones with single copy integration, which were then screened for efficiency of RMCE using a test plasmid. Two clones, HuH7-9 and HuH7-10, showed the mostefficient recombination and were used for integrating plasmids containing regions of the rat *Alb* combined with a GFP reporter (Figs. 1, S1; Table S1).

**Figure 2:**
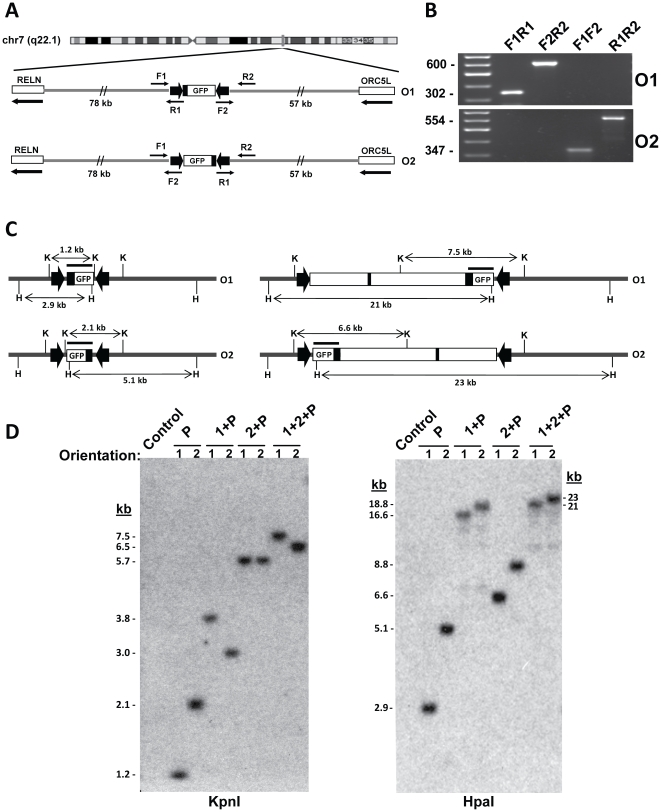
Characterization of integrated constructs. A: Chromosomal location of the targeting cassette in HuH7-9. Maps show representative integration of the 1.2-kb plasmid P. Plasmids may integrate in two orientations (O1, O2) between *RELN* and *ORC5*, respectively, in the same or opposite direction as transcription of both genes (arrows). PCR using primers R1 and F2 (from LoxP), and F1 and R2 (from flanking chromosomal regions), demonstrated that the integrant ends were intact and determined orientation. B: Representative PCR screening of integrants. O1 amplimersare F1 – R1 (302 bp) and F2 – R2 (599 bp). O2 amplimersare F1 – R2 (347 bp) and F2 – R1 (554 bp). C: Representative maps showing predicted Southern blot bands of integrated constructs P and 1+2+P obtained with *KpnI* (K) and *HpaI* (H). The hybridization probe is marked as a bar over the promoter-GFP region, and the promoter and known enhancer are marked in black. D: Southern blot mapping of eight integrated constructs. The blots (left,*KpnI*; right, *HpaI* digests) were probed with a 1.2 kb *FseI* - *SmaI* segment of plasmid P. The expected band sizes are listed in Table S3.

To characterize their transcriptionally-permissive chromosome environments, we sequenced the integration sites of both clones. In HuH7-9, the L1-HYTK-1L cassette was integrated withina 137-kb intergenic region on Chromosome 7, 57 kb from the 3′-end of *Origin of Replication Complex Subunit 5* (*ORC5L*) and 78 kb from the 5′-end of *Reelin* (*RELN*) (Fig. 2A). Both genes are strongly expressed in liver and HCC cells, although neither is specific for these cell types. Surprisingly, the targeting cassette in HuH7-10 was integrated into the same region of Chromosome 7, in this case within the first intron of *RELN*, 116 kb from the 5′-end. This site is 200 kb from the integration site of HuH7-9.

Prior to analysis of gene expression, gancyclovir-resistant isolates were first screened by PCR to demonstrate integration and determine orientation of gene constructs (Fig. 2B, Table S2). Southern blot analysis then discriminated clones with intact single integrations (Fig. 2C, Table S3).

### Analysis of gene expression

**Figure 3:**
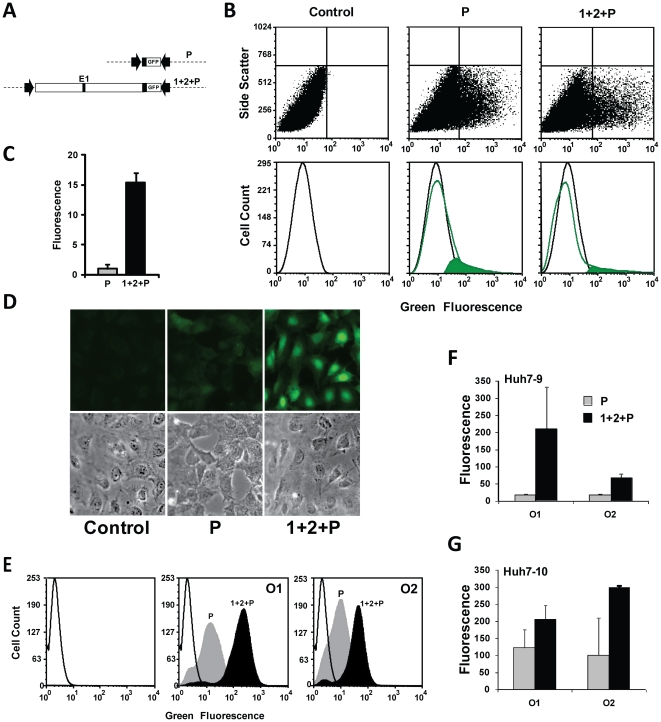
Transient transfection vs. stable gene integration for determining enhancer activity. A: Plasmid constructs. Plasmid P contains the 0.2 kb promoter region fused to GFP. Plasmid 1+2+P also contains a 17.4 kb contiguous upstream segment (Regions 1 and 2). The dotted line represents the vector backbone. B: Transient transfection assays. Upper panel,scatter plots; lower panel, histogram subtraction. The solid green curve is the calculated fluorescence due to transfection. C: Plot of mean fluorescence intensity for each plasmid corrected for molar plasmid concentration and normalized to the promoter value. Average and standard deviation of 3 separate transfection experiments, each with triplicate transfections. D: Cell images. The promoter region produced weak gene expression that was strongly stimulated by the added enhancer region. The images show Orientation 1 integrants. E: Flow cytometric analysis of stable clones showing orientation-dependent stimulation by enhancers. Control cells, open curve; P, gray; 1+2+P, black. F: Expression in HuH7-9 cells. G: Expression in HuH7-10 cells. F, G; Plots show the average and standard deviation of fluorescence from three clones in each orientation. O1, O2; orientations 1 and 2.

To work outanalysis of enhancer function, we studied the albumin promoter alone (plasmid P) or combined with the intact 17.4 kb upstream region (plasmid 1+2+P) that contains well-characterized enhancer E1 (Fig. 3A). Early studies that defined E1 suggested that it functioned poorly in transient assays of large plasmid constructs [25]. Nevertheless, our flow cytometry analysis of transient transfection demonstrated 16-fold enhancement of gene expression (Fig. 3B, C). This result, however, required establishment of conditions that gave a linear measurement of gene expression. Several transient transfection reagents oversaturated gene expression, i.e., they stimulated expression to a plateau level that was insensitive to plasmid concentration or the strength of the reporter gene. In contrast, simple lipofectamine transfection, under conditions that stimulated gene expression in only ∼10% of cells, gave weaker transfection signals that showed a linear relationship between plasmid copy number and level of measured gene expression (data not illustrated).

Transcription oftransiently transfected plasmids is controlled only by regulatory elements within the plasmid construct. In contrast, chromosomal integration allows interaction with transcription control elements outside of the construct. To control for such positional effects, constructs were compared at different integration sites—HuH7-9 or Huh7-10—and in two orientations, designated O1 and O2 (Fig. 3D–G). By flow cytometry, integrants gave a single symmetrical distribution of gene expression with correspondence of mean and peak values.

The promoter and enhancer regions functioned in all integrants. However, expression of the promoter and relative stimulation by enhancers showed significant positional differences. In HuH7-9, the promoter hadsimilar strength in both orientations, while the enhancer region gave 10-fold stimulation in O1 and4-fold in O2. In HuH7-10 cells, the promoter gave 6-fold higher expression than in HuH7-9, with only 2 to 3-fold additional stimulation added by enhancer region. Nevertheless, the combined enhancer and promoter gave gene expression comparable to O1 in HuH7-9 cells. The results in HuH7-10 suggested promoter stimulation by a non-*Alb* enhancer, so subsequent analysis was limited to HuH7-9, comparing O1 and O2 to discriminate positional effects.

### Survey of a 35-kb region for regulatory elements

**Figure 4:**
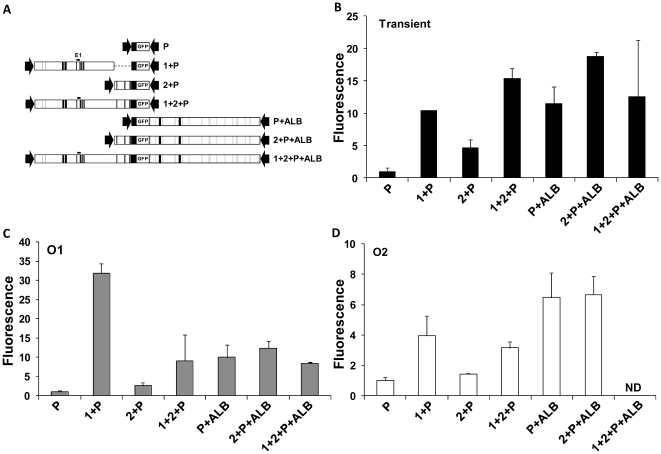
Survey of a 35-kb region for novel regulatory elements. A: Map of plasmid constructs. Conserved noncoding regions, black; *Alb* gene exons, gray; E1, the previously characterized *Alb* enhancer. B: Transient transfection analysis. Mean fluorescence intensity and standard deviation werecalculatedfrom triplicatetransfections in at least three separate experiments. C: Expression of stable integrants in O1. D: Expression of stable integrants in O2. C, D: Mean and standard deviation of expression from at least 2 integrant clones per construct in each orientation, except for a single integrant of 1+2+P+ALB in O1. Fluorescence intensity was normalized to the value obtained for the promoter.

Prior studies localizedE1 from −10.1 to −9.2 kb [25], [27]. Conserved non-coding regions within both the 17.4-kb upstream and the 16.5-kb gene suggested additional regulatory elements (Fig. 1A). To test their transcriptional function and interactions among regulatory elements, we first surveyed large gene regions (Fig. 4).

Transient transfection demonstrated that Regions 1, 2, and ALB each significantly enhanced gene expression, 10-, 4-, and 12-fold, respectively (Fig. 4B). The contribution of Region 2 was additive when combined with either Region 1 or ALB. Transfection of the largest plasmid 1+2+P+ALB (40 kb) was more variable, but its activity was much less than the sum of contributions from individual enhancers.

When integrated, Region 1 and ALB each caused significant enhancement in either orientation (Figs. 4C, D). Gene expression was generally stronger in O1 than O2, particularly stimulation by Region 1. Region 2 had complex effects. It was a weak enhancer by itself, and its small contribution probably added to stimulation by ALB. However, Region 2 inhibited stimulation by Region 1, especially in O1.

The largest plasmid construct, 1+2+P+ALB, combined all of the regulatory regions.Its large size led to low efficiency of integration, and we obtained only a single integrant, in O1. The combined stimulation by Regions 1, 2 and ALB in this clone was not greater than individual component segments, an effect also noted in transient expression.

Thus, transient transfection and stable integration in either orientation demonstrated enhancer function. However, genomic integration revealed complex relationships among regulatory elements within the constructs, andpositional effects suggested interaction with additional elements outside of the constructs.

### Mapping of novel enhancers

**Figure 5:**
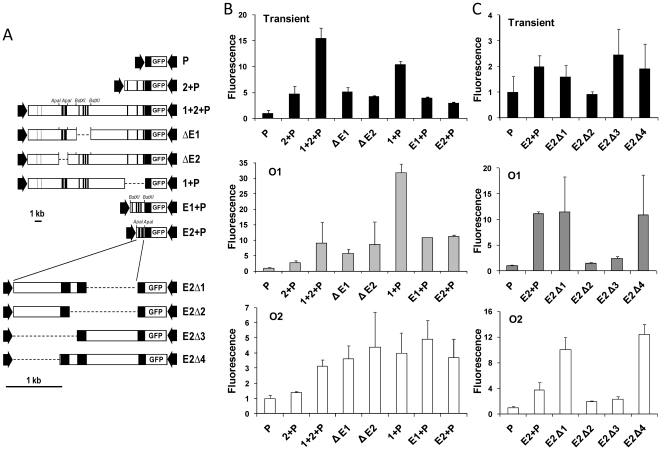
Localization of E2, a novel upstream *Alb* enhancer. A: Maps of deletion constructs. Large deletions of 1+2+P (top) were constructed with restriction enzymes *ApaI* and *BstXI*. Plasmid E2+P was further deleted by PCR (bottom; see Table S2). Black, regions highly conserved between rat and human genes. B: Analysis of plasmids containing intact enhancers. The plots show analysis of transient assays and integrated constructs in two orientations (O1, O2). C: Analysis of plasmids for fine mapping of E2, as in panel B. For each construct, mean and standard deviation of fluorescence intensity were calculated from 3 separate transient transfection experiments, or from multiple integrant clones.

The regional analysis and pattern of sequence conservation both suggested enhancers throughout the 35-kb region. We first focused on Region 1. Deletion mapping—with constructs analyzed by both transient transfection and stable integration—identified a new enhancer, E2 (Fig. 5). E1 and E2 were of similar strength and together accounted for all of the enhancing activity within Region 1. The two enhancers functioned in their normal positions or when moved close to the promoter, and their enhancer function was independent of each other and of Region 2. Deletion mapping localized E2 to the −12.8 to −11.9 interval that contained 2 regions of conserved sequence. In stable integration, both regions were required for enhancer function, although only the more proximal conserved region was required for enhancer function in transient assays. By itself, Region 2 (3.7 kb) acted as a weak enhancer (“E3”) in transient assays, where it added to the stimulation by Region 1. However, deletion mapping did not discretely localize an enhancer within Region 2 (not illustrated). Moreover, in stable integration, Region 2 had an inhibitory effect when combined with Region 1.

**Figure 6:**
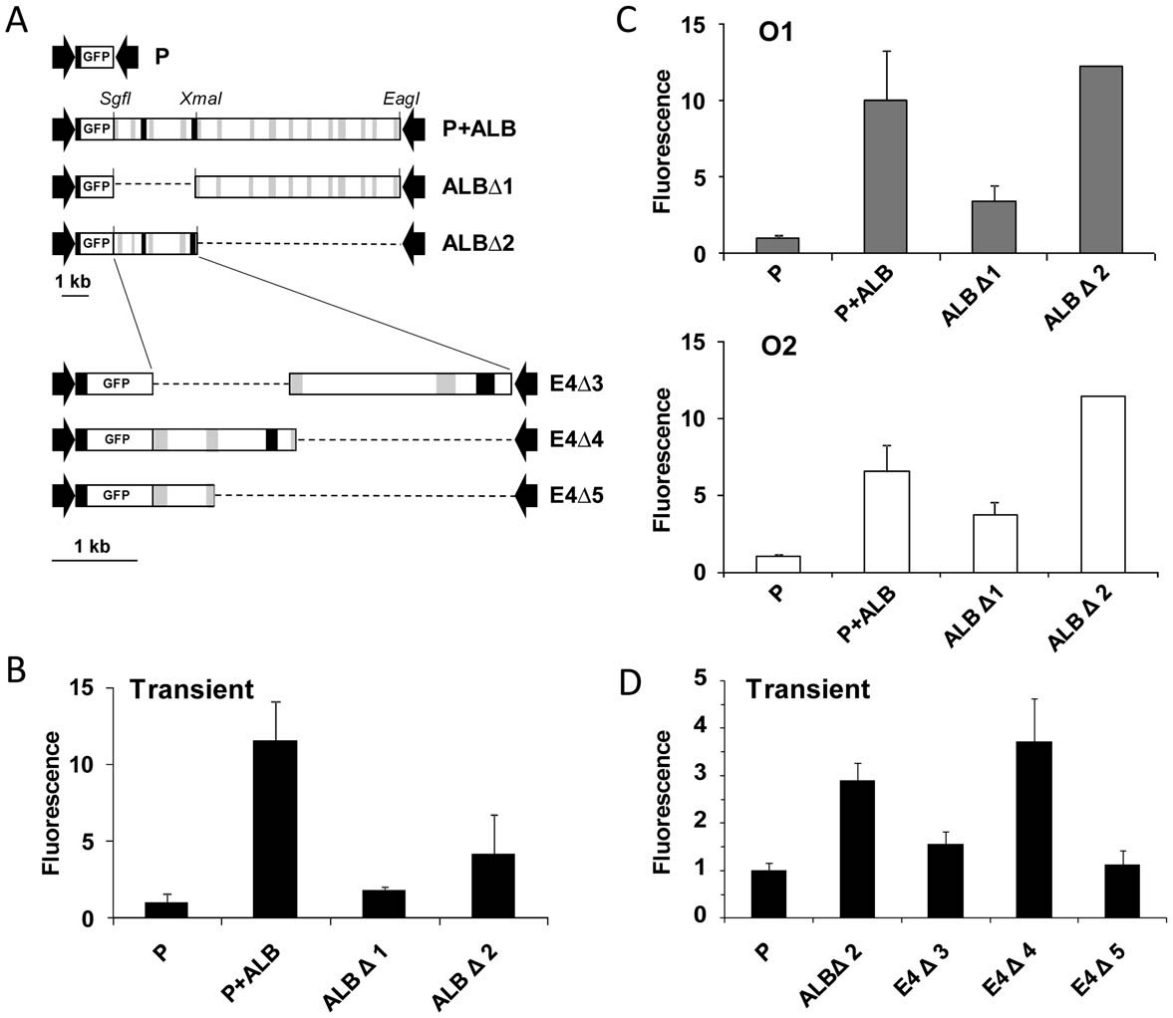
Localization of intronic enhancer E4. A: Large deletions clones of P+ALB were constructed with restriction enzymes *SgfI*, *XmaI*, and *EagI* (top). Clone ALBΔ2 was then further deleted by PCR (bottom; see Table S2). B: Analysis of the larger constructs by transient transfection. C: Analysis of the larger constructs by integration in two orientations (O1, O2). D: Localization of a strong enhancer toIntron 2 by transient transfection assays. B, D; Mean and standard deviation of fluorescence intensity were calculated from two transient transfection experiments. C; Average and standard deviation of measurements from integrated constructs.

The 16.5-kb *Alb* gene itself caused strong transcriptional enhancement, and contains conserved noncoding regions within introns 2 and 4 (Fig. 6). Deletion mapping, analyzed by both transient transfection and stable integration, localized a strong enhancer in a proximal 4.4-kb segment. Analysis of additional deletions by transient transfection localized the strong enhancer to intron 2. Two other segments, within pALBΔ1 and pE4Δ3, mediated weak enhancement, but the responsible elements were not further resolved.

### Modification of gene expression

We next evaluated the effects of growth arrest on enhancer-driven gene expression. Because it is likely that DNA replication and cell division will disrupt enhancer-promoter interaction, the expectation was that gene expression would increase when the cell does not divide, and the magnitude of the effect will depend on how long it takes to reestablish looping. This is consistent with two observations. Constitutively proliferating HuH7 cells strongly express albumin mRNA, but at 40% of the level of normal liver [34], while *Alb* mRNA in normal liver decreases significantly when hepatocytes are stimulated to proliferate by partial hepatectomy [35].

HuH7 is a contact-inhibited cell line, so we compared growth arrest induced by confluenceor serum starvation. Analysis showed that HuH7 cells arrested at 60–72 hr after plating at 25% confluence, or 24 hr after serum starvation (data not illustrated).

**Figure 7:**
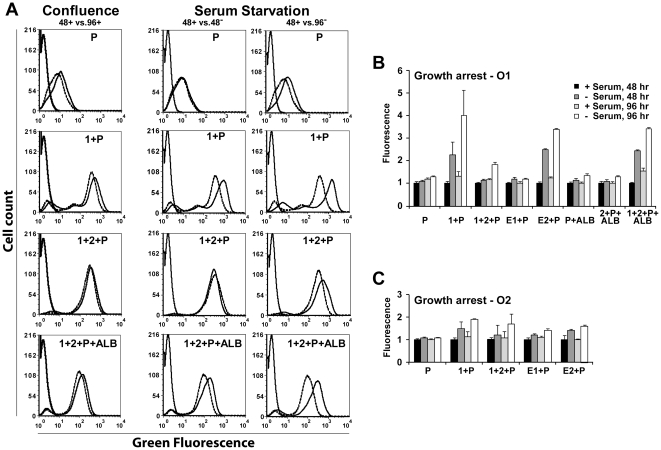
Effects of growth on enhancer function. A: Selected flow cytometry comparisons. Cells were plated at 25% confluence in 5% fetal calf serum, and allowed to reach confluence (∼72 hr) or switched to 0.1% serum after 24 hr. Exponential growth at 48 hr in serum-containing medium (dotted lines) was compared to growth arrest (solid lines) due to cell confluence at 96 hr (left), or serum starvation at 48 and 96 hr (center and right). A control curve for untransfected cells appears at the left of each panel (solid lines). B: Stimulation of O1 clones by growth arrest. A series of clones systematically resolved the effects onintact Regions 1, 2, ALB, and Enhancers E1 and E2. C: Stimulation ofO2 clones by growth arrest. A more limited series of O2 clones was evaluated as above. B, C; the plots show mean and standard deviation of fluorescence intensity averaged from two separate experiments. Expression was normalized to the level observed for growth at 48 hr in serum.

The two arresting conditions had significantly different effects on gene expression (Fig. 7). Confluence caused a moderate increase, less than 2-fold, in the expression of two constructs, 1+P and 1+2+P+ALB. In contrast, serum starvation stimulated all Orientation 1 integrants to some extent, up to 4-fold by 96 hr. Strong stimulation was observed in long constructs 1+P and 1+2+P+ALB, and in short construct E2+P, and was thus selective for E2. The presence of Region 2 had an inconsistent effect. It reduced stimulation of 1+2+P compared to 1+P, but did not affect stimulation of 1+2+P+ALB. Stimulation of Orientation 2 clones was more moderate, with little selectivity for E2. The difference between confluence and serum starvation could reflect that the latterarrests growthmore effectively, or alternatively, that serum contains a factor that selectively inhibits E2.

## Discussion

We set out to create an experimental system of gene integration into a controlled chromosomal location with the eventual goal of resolving specific molecular interactions within a complex locus. The model of gene expression was *Alb*, a distinctive high-level phenotypic marker of hepatocytes. Our analysis exploited the hepatocyte-like phenotype of *Alb*-expressing HuH7 cells, where function of *Alb* transcriptional regulatory elements is similar—though perhaps not identical—to normal hepatocytes.

**Figure 8:**
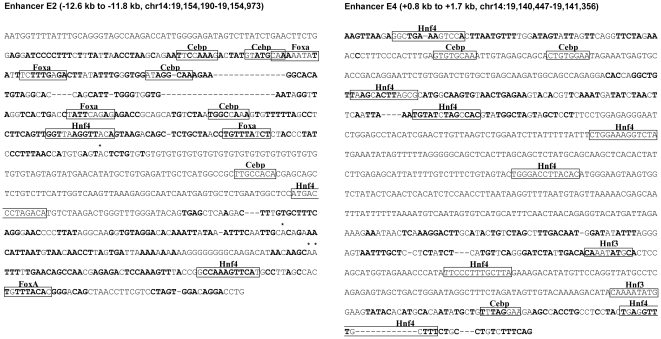
Sequence analysis of two new Alb enhancers. Left: E2, localized to a 784 bp region by deletion mapping. Right: the 974 bp minimal region containing E4. Both regions were aligned to the human sequence (DCODE, http://ecrbrowser.dcode.org/); bold, conserved bases; *, insertion in the rat gene; -, insertion in the human gene. Putative transcription factor binding sites were detected with the TRANSFAC database using the RVISTA utility of DCODE, and with binding site models compiled in our laboratory. Sites with high-rank matches by both analyses were annotated in the figure. The ENCODE tracks at the human Alb gene, based on the Human Mar. 2006 (NCBI36/hg18) Genome Assembly (http://genome.ucsc.edu/cgi-bin/hgTracks), include studies of HepG2 cells which mapped chromatin hypersensitive regions and ChIP localizations of HNF4 and C/EBP binding. For both enhancers, the human regions collinear with the illustrated rat sequences have hypersensitive regions and bind HNF4 and C/EBP, although these localizations cannot be precisely mapped to individual binding sites in either genome.

The analysis validated the gene targeting system by demonstratingtwo novel *Alb* enhancers, E2 and E4. The weak activity of the previously characterized E1 has seemed inadequate to explain the very strong expression of *Alb* in hepatocyte-derived cells, so the new observations eliminate a significant discrepancy. Comparative sequence analysis showed that E2 and E4 have features typical of hepatocyticenhancers, with putative sites for their characteristic binding factors, HNF4, FOXA/HNF3, and C/EBP (Fig. 8). Moreover, ENCODE databases derived from analysis of HepG2 cells showed that the human counterpart regions had hypersensitive sites and bound both HNF4 and C/EBP (http://genome.ucsc.edu/cgi-bin/hgTracks). Phylogenetic comparisons demonstrated that enhancers have conserved regions flanked by specific binding sites that may be poorly conserved [36], [37]. Nevertheless, enhancers from different species tend to bind the same transcription factors near the conserved regions, sometimes in different positions. Thus, prediction of transcription factor binding sites and genomic analysis of human counterpart regions are both highly consistent with our functional characterization of these enhancers.

Prior RMCE studies have analyzed orientation-dependent effects of flanking sequences on transgenes, position-dependent silencing of the β-globin LCR, and transcriptional interference at a complex locus [33], [38], [39]. We adapted RMCE to examine enhancer-mediated regulation over long distances. Comparison of integrated constructs with transient transfection showed that each method has limitations: transient assays give a simplified view of gene expression, but integratedconstructs can interactwith unrelated regulatory elements. The latter effects, however, can be constant if integration site, orientation, and copy number are eliminated as experimental variables. Both transient and RMCE analyses revealed that Region 1 and ALB mediate independent and comparable enhancement of gene expression. However, the behavior of smaller regions and the intact Region 2 exemplifies the differences between transient and integrated regulatory elements.By itself, Region 2 functioned as an enhancer in transient assays, where it caused 5-fold stimulation of gene expression and was additive with either Region 1 or ALB. Moreover, Region 2 had been previously shown to contain a hypersensitive site [25]. We therefore considered Region 2 to contain a third enhancer (E3), which we did not resolve further because of itsweak activity. Integrationof Region 2 gave a different perspective, since it produced little transcriptional stimulation by itselfand reduced stimulation by Region 1 in larger constructs. Thus Region 2 contains regulatory elements, but their specific function and contribution to *Alb* gene expression require further investigation.

Another difference between transient and integrated expression was revealed by deletion mapping of E2, which was localized to an 0.8 kb genomic interval that contains two conserved regions.The E2Δ3 construct eliminated one conserved region and still functioned as an enhancer in transient assays. In contrast, enhancer function after integration required both conserved regions, revealing an essential activity that is dispensable for transient expression from a plasmid.

Transfection of the largest plasmid, 1+2+P+ALB, was challenging due to its size and low copy number, but even this large plasmid functioned in transient assays at a level indicating enhancer-stimulated gene expression. Both transient assay and chromosomal integration produced intermediatereporter stimulation, indicating that the enhancer regions were not additive or synergistic. The level was much less than the maximum capacity of the promoter since the 1+P construct was about 3-fold stronger. Because each can separately stimulatetranscription, the lack of additive stimulation suggestedenhancer competition. This could result from competition for binding to the same element within the promoter or competition for a limiting trans factor. Alternatively, the effect could represent binding of the enhancers to each other in competition with their binding of the promoter, or an indirect boundary function where one enhancer establishes a local chromatin structure that obstructs access of the other to the promoter. Any of these functions might modulate during development, so they are all possible mechanisms for enhancer switching.

Transgenic studies of the β-globin gene cluster and its LCR led to the concept that position independence is an ideal criterion for demonstrating that a regulatory region is intact [24]. In this paper, however, we demonstrated that an extended region of the rat *Alb* locus does not show complete position independence. Perhaps additional elements outside the 35 kb region contribute to higher-order *Alb* regulation [40]. Alternatively, position-independence may be an artificial concept, since each gene has been selected to function in its natural position, surrounded by local and distant regulatory elements. From another perspective, demonstration of position effects in our system has revealed functional cis elements that are obscured by simpler assays of gene expression.

It is well established that promoters and enhancers loop together, but looping represents different kinds of interactions. The distant LCR dynamically oscillates between β- and γ-globin genes by the distant LCR, transitory looping within a more stable chromatin hub [41], [42], [43]. In contrast, the HNF4α-gene promoter and enhancer require two days to form a stable loop after growth arrest of Caco2 cells [44]. In either type of looping, the higher order chromatin architecture will be disrupted by DNA replication. We therefore investigated whether growth arrest increased the effects of enhancers, particularly synergy of multiple enhancers in the full 35-kb *Alb* region. Natural *Alb* expression is consistent with these observations, because proliferating hepatocytes or cell lines have lowerexpression than the quiescent hepatocytes of normal liver [34], [35]. Indeed, growth arrest strongly stimulated gene expression, particularly of constructs containing E2. The next stepsare to work out the mechanism of this specific effect, along with a general characterization of the dissolution and reassembly of looping during the cell cycle. Our system of targeted integration and *Alb* gene constructs will provide an ideal platform for this research.

## Materials and Methods

### Plasmid constructs

Cloning vectors Locus Linker 1 and 2 (pLL1, pLL2) were constructed for assembly of restriction enzyme fragments excised from genomic clones of the rat *Alb-Afp* locus, and for Cre-mediated recombination into mammalian cells (Fig. 1, Table S1). A 290 bp synthetic linker (Figs. 1B and S1) was inserted between the *NdeI* and *HindIII* sites of pUC19 (pLL1) and also transferred to pBR322 (pLL2). The low copy number of the latter facilitates cloning of large genomic DNA segments. The plasmids incorporate the following features. Two loxP sites in opposite orientation allow Cre-mediated insertional recombination with the targeting cassette. Two I-SceI sites flank the cloning linker to allow incision of integrated gene segments. Unique restriction sites that do not cut the *Alb-Afp* locus (*BssHII*, *FseI*, *SgfI*, *BsiWI*, *SgrAI*, and *SfiI*), or cut the entire locus only once (*SmaI*, *EagI*, and *SacII*), were arranged so that individual regions could be joined or excised through simple forced directional cloning strategies. Other restriction enzyme sites were arranged to facilitate locus assembly or to provide general cloning sites.

*GFP* was amplified by PCR from pEGFP-N1 (Clontech, Mountain View, CA) with primers that added terminal *SgfI* and *SmaI* sites (Table S2) and cloned into pLL1 at those sites. The synthetic Alb123 promoter [45] was inserted between linker *PstI* and *BglII* sites.

For cloning of large DNA segments, restriction fragments were resolved on 0.5% agarose gels stained with SYBR gold (Invitrogen, Carlsbad, CA), detected with a Dark Reader Transilluminator (Clare Chemical Research, Denver, CO), and purified using a QIAEX II gel extraction kit (Qiagen, Hilden, GER). Ligations, performed with standard procedures, were electroporated into DH10B (Invitrogen) or SURE (Stratagene, La Jolla, CA). Deletion mapping of enhancer-containing regions was carried out with *BstXI*, *ApaI*, *SgfI*, or *SmaI*, or withspecific PCR primers (Table S2).

### Cell clones for targeted integration

A 2.5 kb *PvuII* fragment containing the cassette pL1-HY-TK-1L [32] was electroporated into HuH7 [34], [46]cells and selected with 200 µg/ml of Hygromycin B. Hyg^R^ colonies were maintained under continuous antibiotic selection. Southern blot analysis (not illustrated) demonstrated fourclones that contained single cassette copies. These were screened by transfection of pL1-CMV-Neo-1L alone or in cotransfection with pCMV-Cre/pBS185 [47], followed by selection with geneticin. The number of colonies was counted in each condition, and the difference between the two values represented the number of Cre-targeted integrations. Among the four clones, targeted integration ranged from 29–78 targeted integrations per 10^6^ cells. HuH7-9 had the greatest efficiency of targeted integration and was used as the standard system for analysis of albumin gene constructs after an initial comparison with HuH7-10 (see Fig. 3 below).

### Cell culture and transfection

HuH7-9 and HuH7-10 cells were maintained in Williams E medium supplemented with 1% L-Glutamine, 1% Pen/Strep, and 5% fetal bovine serum at 37°C in 5% CO2. For transient assays, 0.5×10^6^ cells were transfected with 2.5 µg of DNA in Lipofectamine (Invitrogen) according to the manufacturer's protocol. Cells were fed with fresh medium at 24 hr, then trypsinized, washed, and analyzed by flow cytometry at 48 hr. For targeted integration, cells were reselected with 350 µg/ml hygromycin B solution for one or two passages before stable transfection. Then 1×10^6^ cells were transfected with 4 µg of gene constructs in pLL1 or pLL2 and 1 µg pCMV-Cre (Clontech), using Lipofectamine LTX with PLUS reagent (Invitrogen), on 60 mm plates. At 24 hr, cells were split 1∶2 to 100 mm plates. 1 µM gancyclovir was added at 48 hr [33]. Discrete colonies with uniform fluorescence were isolated after 3–4 weeks of selection. Isolates were screened by PCR and Southern blots analysis [48].

To study the effects of growth arrest, control and GFP-expressing cell clones were seeded at low density (∼25% confluence) and fed with Williams E medium containing 5% or 0.1% serum 24 hr after plating. Clones were analyzed by flow cytometry 48 and 96 hr following the change of medium.

### Genomic location of the targeting cassette

Genomic DNA was digested with *AvrII* or *PvuII* and self-ligated at 1–2 ng/µl. Inverse PCR of the cassette-genomic junctions used primers Target F and Target R. PCR products (1–4 kb) were sequenced using the same primers.

### Flow Cytometry

1–2×10^6^cells were trypsinized, washed, resuspended in cold PBS containing 1% FBS, and analyzed with a FACSCalibur flow cytometer (Becton-Dickenson, Franklin Lakes, NJ). Forward and side scatter measurements were used to differentiate live and dead cells. Measurements for 20,000 cell events in stable clones, or 60,000 cell events in transient transfection assays, were further analyzed with FCS Express software (De Novo Software, Los Angeles). For transient assays, average fluorescence intensity was determined by histogram subtraction of the distribution determined for untransfected cells from distribution of transfected cells. Since all transfections utilized the same DNA concentration of transfected plasmid, the mean fluorescence was then corrected for relative plasmid copy number in each transfection. For stable clones, values determined were mean fluorescence intensity, and position of the peak of highest gene expression. In most cases, at least two separate clones were analyzed in each orientation.

## Supporting Information

Figure S1 **Linker for assembly of the Alb-AFP gene region.** The linker was cloned into NdeI and HindIII sites of pUC19. Restriction enzyme sites in large bold type were used for assembly of rat Alb and AFP gene segments. L1 and 1L are loxP sites in opposite orientations.
(DOCX)

Table S1 Plasmids.
(DOCX)

Table S2 PCR primers.
(DOCX)

Table S3 Bands detected by Southern blot.
(DOCX)
