# Supplementary material for: Systematic Targeted Integration to Study Albumin Gene Control Elements
Source: PLoS One. 2011 Aug 12;6(8):e23234. doi: 10.1371/journal.pone.0023234 (PMC3155544; doi:10.1371/journal.pone.0023234)
Supplement: Figure S1 — Linker for assembly of the Alb-AFP gene region. The linker was cloned into NdeI and HindIII sites of pUC19. Restriction enzyme sites in large bold type were used for assembly of rat Alb and AFP gene segments. L1 and 1L are loxP sites in opposite orientations. (DOCX) [file pone.0023234.s001.docx]

**Figure S1. Linker for assembly of the Alb-AFP gene region.** The linker was cloned into NdeI and HindIII sites of pUC19. Restriction enzyme sites in large bold type were used for assembly of rat Alb and AFP gene segments. L1 and 1L are loxP sites in opposite orientations.
