# Supplementary material for: Systematic Targeted Integration to Study Albumin Gene Control Elements
Source: PLoS One. 2011 Aug 12;6(8):e23234. doi: 10.1371/journal.pone.0023234 (PMC3155544; doi:10.1371/journal.pone.0023234)
Supplement: Table S1 — Plasmids. (DOCX) [file pone.0023234.s002.docx]

| **Table S1. Plasmids** | |  |  |
| --- | --- | --- | --- |
|  | **Construct** | **Vector** | **Plasmid (kb)** |
| **Cloning vectors** | | | |
| pLL1 | Linker | pUC19 | 2.7 |
| pLL2 | Linker | pBR322 | 4.8 |
| **Master plasmids** | | | |
| Alb123 | Synthetic *Alb* promoter (0.2 kb) | LL1 | 2.9 |
| Region 1 | -17.6 to -3.9 kb | LL1, LL2 | 16.4, 18.5 |
| Region 2 | -3.9 to -0.2 kb | LL1 | 6.4 |
| ALB | *Alb* gene (0 to + 16.5 kb) | LL1 | 19.2 |
| **Reporter plasmids** | | | |
| P | Alb123 + GFP | LL1 | 3.9 |
| 2+P | Region 2 + P | LL1 | 7.5 |
| 1+P | Region 1 + P | LL2 | 19.5 |
| 1+2+P | Regions 1 + 2 + P | LL2 | 23.2 |
| P+ALB | P + ALB | LL1 | 20.4 |
| 2+P+ALB | Region 2 + P + ALB | LL1 | 24.1 |
| 1+2+P+ALB | Regions 1 + 2 + P + ALB | LL2 | 41.8 |
| E1+P | (-10.1 to -7.1) + P | LL1 | 6.9 |
| E2+P | (-13.4 to -11.0) + P | LL1 | 6.3 |
| **Reporter plasmids with deletions** | | | |
| ALBΔ2 | P + (0 to +4.4) | LL1 | 8.3 |
| ALBΔ1 | P + (+4.4 to +16.5) | LL1 | 16.0 |
| ΔE1 | (-17.6 to -13.4) + (-7.1 to -0.2) + P | LL2 | 19.4 |
| ΔE2 | (-13.4 to -11.9) + (-11.0 to -0.2) + P | LL2 | 20.8 |
| E2Δ1 | (-13.4 to -11.9) + P | LL1 | 5.4 |
| E2Δ2 | (-13.4 to -12.4) + P | LL1 | 4.9 |
| E2Δ3 | (-12.2 to -11.0) + P | LL1 | 5.1 |
| E2Δ4 | (-12.8 to -11.0) + P | LL1 | 5.7 |
| E3Δ1 | (- 3.9 to - 2.0) + P | LL1 | 5.9 |
| E3Δ2 | (- 2.0 to -0.2) + P | LL1 | 5.7 |
| E3Δ3 | (-0.9 to -0.2) + P | LL1 | 4.9 |
| E4Δ3 | P + (+1.8 to + 4.4) | LL1 | 6.4 |
| E4Δ4 | P + (+1.0 to + 2.2) | LL1 | 5.7 |
| E4Δ5 | P + (+1.0 to + 0.9) | LL1 | 4.7 |
