# Supplementary material for: Systematic Targeted Integration to Study Albumin Gene Control Elements
Source: PLoS One. 2011 Aug 12;6(8):e23234. doi: 10.1371/journal.pone.0023234 (PMC3155544; doi:10.1371/journal.pone.0023234)
Supplement: Table S2 — PCR primers. (DOCX) [file pone.0023234.s003.docx]

**Table S2. PCR primers**

| **Oligo Sequence** |
| --- |
| **Screening of stable clones at the targeting locus**  **F1**  CATTATTTGCTTTTATTTTATAGTGTTTGTGCTC  **F2** GGGATAACAGGGTAATCCCATAACTTCGT  **F3** GCAAGGCGATTAAGTTGGGTAACG  **R1** CGCATTACCCTGTTATCCCTAGATATAACTTC  **R2** CAGCATATCACAGTATCTAAGTGCTAGTTACAGG  **R3**  GCGGGCAGTGAGCGCAAC  **Inverse PCR and sequence of flanking regions**  **Target F** GGGTGCCTAATGAGTGAGCTAACTCACA  **Target R** CGTTACCCAACTTAATCGCCTTGC |
| **Mapping of E2**  **E2 F1** CATCGATCTGCAGCTTCAGATGGC  **E2 F2** TGGACGAGCTACTCCGTGTATCTCTCTG  **E2 F3** GGGTTTGGGATACAGTGAGCTCAAGAC  **E2 R1** AAGACCGTGTTATCATAAGCAACAACAGG  **E2 R5** CAAGATTCTTACTGCTGGGAATAATGATAATGC  **E2 R6** AGGGATAGGGTAGAGATAAACAGGTTAGCAG  **E2 R7** CAACCAGGTCCTGTCCACTAGGACG |
| **Mapping E3** |
| **E3 F1** ACAAAGCCTTTGGGGGGTTGG  **E3 F2** TGACCATCCCAGCTATTTTCTATTCGG  **E3 F3** TTCCATTTCCACTATGAGGTTTTGGC  **E3 R1** GTTTCCGAATAGAAAATAGCTGGGATGG  **E3 R2** GGGTATAGAAAATGGACTCCTTTATTCGG |
| **Mapping E4** |
| **E4 F1** GAGTCCTGATTGCCTTTTCCCAGTATC  **E4 F2** AACAAGAGACAAAGACCATCACTCCCC  **E4 R1** GTTACCCACTTCATTGTGCCAGAGGC  **E4 R2** CAATGGACTTGTCACAGTTTTCGGC  **E4 R3** AGGCCTTTGAAATGCTGTTCTCCTAAG |
