# Supplementary material for: Systematic Targeted Integration to Study Albumin Gene Control Elements
Source: PLoS One. 2011 Aug 12;6(8):e23234. doi: 10.1371/journal.pone.0023234 (PMC3155544; doi:10.1371/journal.pone.0023234)
Supplement: Supplementary file 4 [file pone.0023234.s004.docx]

Table S3. Bands detected by Southern blot

**Restriction Enzyme:**	***KpnI***		***HpaI***
**Orientation:**	**O1**	**O2**	**O1**
**<u>Construct</u>**			
P	1.2 kb	2.1 kb	2.9 kb
1+P	3.8	3.0	16.6
2+P	5.7	5.7	6.6
1+2+P	7.5	6.7	20.7
P+ALB	18.6	18.6	2.9
2+P+ALB	22.3	22.3	6.6
1+2+P+ALB	24.0	23.1	20.7
ALBΔ2	6.5	5.6	2.9
ALBΔ1	14.6	14.6	2.9
E2Δ1	2.9	3.8	7.2
E2Δ2	2.5	3.4	6.7
E2Δ3	2.7	3.6	6.9
E2Δ4	3.3	4.2	7.5
